# Comparison Between Larval Survey Index and Positive Ovitrap Index in the Evaluation of Populations of *Aedes* (*Stegomyia*) *aegypti* (Linnaeus, 1762) North of Paraná, Brazil

**DOI:** 10.1177/1178630219886570

**Published:** 2020-01-06

**Authors:** Kauani Larissa Campana Nascimento, João Fernando Marques da Silva, João Antonio Cyrino Zequi, José Lopes

**Affiliations:** 1 Programa de Pós-graduação em Ciências Biológicas, Departamento de Biologia Animal e Vegetal, Centro de Ciências Biológicas, Universidade Estadual de Londrina, Londrina, Brazil; 2 Departamento de Biologia Animal e Vegetal, Centro de Ciências Biológicas, Universidade Estadual de Londrina, Londrina, Brazil

**Keywords:** Dengue, mosquito vectors, oviposition

## Abstract

*Aedes* (*Stegomyia*) *aegypti* (Linnaeus, 1762) is one of the world’s most widely distributed mosquitoes and is the vector of the dengue virus, one of the most important reemerging diseases. Besides dengue, *A. aegypti* can also transmit urban yellow fever, chikungunya, and Zika virus, making it of great medical importance. Thus, it is of extreme importance to find reliable methods to evaluate the presence of *A. aegypti* in urban areas. In Brazil, rapid index surveys of *Aedes aegypti* by means of larval survey (LIRAa) is the official method to estimate the Breteau (BI) and property infestation (PII) indexes, which indicates how many infested containers with larvae of *A. aegypti* were found by the total number of properties surveyed and the proportion of houses infested, respectively. As the LIRAa requires access to private residences and trained personal to find breeding sites and do not reveal the mosquito’s presence when in low density, it has not demonstrated efficacy in determining the presence of *A. aegypti*. To evaluate an alternative method, the LIRAa method was compared with an oviposition trap, made with hay infusion and a hardboard pallet, to evaluate the BI and the PII. The 2 methods were carried out simultaneously through 4 surveys, sampling 60 homes per survey. To evaluate the best configuration of ovitraps for surveillance of *A. aegypti*, the ovitraps were installed in intradomicile and peridomicile areas, with 1 to 5 traps per residence and with 1 to 3 pallets per trap, and these different configurations were compared using the positive ovitrap index (POI) and egg density index (EDI). The ovitraps showed greater sensitivity for detecting the presence of *A. aegypti*, with a BI of 72.5% and PII of 54.2%, whereas the LIRAa revealed only 2.1% for the BI and 1.3% for the PII. Therefore, the use of sentinel traps can provide information in a more rapid and precise manner. As there were no differences in the ovitraps distributions patterns, the ovitraps can be installed in the peridomicile area, with 2 traps per surveillance point and 1 pallet per trap, making their installation easier and more cost-efficient, facilitating the work of health agents in future surveillances complementing LIRAa’s actions for efficient monitoring.

## Introduction

Dengue is an arbovirosis that appears to be one of the most important reemerging diseases in the world.^1^ The causal agent of the disease is an arbovirus of the genus *Flavivirus*, belonging to the family Flaviviridae, with 4 known sorotypes: DENV-1, DENV-2, DENV-3, and DENV-4. Humans are the source of infection and the vertebrate reservoir.^1^ Some mosquitoes in the genus *Aedes* are the vectors of the etiological agent, where *Aedes* (*Stegomyia*) *aegypti* (Linnaeus, 1762), the most important in transmission, can also cause urban yellow fever.

In Brazil, in recent years, *A. aegypti* gained more prominence after it was proven that in addition to dengue, the mosquito still transmits chikungunya and Zika virus, registering the first cases in 2014^2^ and 2015,^3^ respectively. Confirmation of autochthonous transmission occurred in the country as of April 2015, and 18 states with the virus.^4^ *A. aegypti* can use artificial breeding sites for oviposition, originating from anthropic activity, represented mainly by containers that allow the accumulation of water and are located around or inside homes,^5,6^ and expanded throughout the national territory and affecting increasing numbers of people each year.^4^ Díaz-González et al^7^ verified that about 22% of the captured *A. aegypti* present infection by the chikungunya virus. The microcephaly relationship caused by Zika virus in the northeast region of the country was established. The cases of microcephaly in Brazil were 1434 cases confirmed according to data from the epidemiological report up to May 21, 2016.^8,9^

*Aedes aegypti* is the exclusive vector of dengue virus and of urban yellow fever virus in Brazil.^10^ Since 1986, from a large epidemic that reached the metropolitan region of Rio de Janeiro, cases of dengue are continuously registered in almost all Brazilian states. This expansion of dengue fever reached national proportions thanks to the rapid spread of *A. aegypti*.^11^

Therefore, dengue is featured as the most important arbovirosis in the world, with approximately half of the global population at risk, where 50 to 100 million cases per year are estimated, in more than 100 endemic countries, which corresponds to an increase of more than 30 times the number of cases registered annually during the last 50 years.^1^ This marked increase has been of concern for society and health authorities, due to the difficulties with the control of epidemics and care of individuals affected by dengue.^12^

To evaluate the presence and abundance of *A. aegypti* and *Aedes albopictus*, the Ministry of Health of Brazil carries out larval surveys as an official method, where the Breteau and property indices are obtained.^13^ *A. albopictus* is abundant in areas with vegetation near reside, also known as periurban areas.^6^ Vega-Rúa et al^14^ verified that both species, *A. aegypti* and *A. albopictus*, were able to transmit all 3 genotypes of chikungunya and reached alarming rates in Asia and Africa, thus the spread and establishment of chikungunya in the Americas presents as an imminent risk. The indices take into consideration the number of homes visited and the presence or absence of larvae.^15^ Even having operational difficulties, such as access to residents’ homes, locating the cryptic breeding site such as gutters, tree holes among others, the results obtained by these indices guided the control of the vector.

The principal method in the control of *A. aegypti* in Brazil is the elimination of previously determined infestation. The health agents of endemics pay periodic visits to urban buildings, where they locate and eliminate breeding sites; they use larvicides or adulticides for the reduction of the vector.^15^ In this strategy of eliminating breeding sites, little efficacy was observed, because in Brazil, there is a growing number of epidemics of dengue in all the country.^16^ In 2011, about 764 032 cases of dengue were registered in Brazil, of which 35 438 cases were in Paraná.^17^ According to the Ministry of Health, in 2012, in Brazil, there were about 591 384 cases of dengue, where 5398 cases were in the state of Paraná. In 2019 Brazil recorded 1,439,471 probable cases of dengue between the epidemiological weeks (1 to 34); an increase of 599.5% over the same period last year.^55^

The larval surveys are questioned as the method of evaluation of *A. aegypti*, because when the levels of the infestation of the vector are low, they do not reveal its presence.^13^ Thus, the use of alternative and additional methods has become necessary. The use of traps for oviposition (ovitraps) is a method of collecting eggs that provides an evaluation of the density of *A. aegypti*, because they attract gravid females for oviposition, also making it possible to eliminate eggs from the environment.^18-20^

Ovitraps appear to be efficient in studies for the detection of *Aedes*,^6,21-24^ because they detect the vector at low densities; it is also a more economic method than larval survey.^23,25,26^ Chadee et al,^27^ in studies with ovitraps in the peridomicile area, found that about 80% of eggs collected were on the pallets (flat and rough wood blade on 1 side) inside the ovitraps tested. Focusing on using ovitraps in field studies aimed at corroborating the data on the reproductive biology of *A. aegypti*, the objectives of this work were to study aspects of the oviposition of this vector, to compare the indices obtained using the ovitraps with the larval survey, to determine the number of sentinel ovitraps necessary per home, to evaluate the ideal number of pallet per trap for determining a reliable determination of infestation by *A. aegypti*, and to find the best location for the installation of ovitraps in homes for surveillance actions and control.

## Material and Methods

### Study area

The study was conducted in the urban area of the municipality of Cambé, Paraná, Brazil, in homes located in the neighborhoods of Ana Rosa (divided into 2 areas: Ana Rosa 2 and Ana Rosa 3), Cambé 4, and Jardim Santa Isabel. The municipality of Cambé-PR, located at (51°15′37.44″ at 51°15′11.52″W and 23°16′0.48″ at 23°16′26.4″S), with an average altitude of 650 meters, has a humid mesothermic subtropical climate, with warm and rainy summers (mean maximal temperature of 22°C), mild winters (mean minimal temperature below 18°C), and without defined dry season.^28^ The area was chosen due to the relevant presence of *A. aegypti* with the latest LIRAa surveys.

### *Sampling for the Rapid Index Survey for Aedes aegypti*—(*LIRAa*) *and ovitraps*

For comparison of the indices, we monitored 4 surveys conducted by health agents of the Municipal Secretariat of Health of the Municipal Prefecture of Cambé, in the above described neighborhoods, in the following periods: November 28 to December 2, 2011; January 9 to January 13, 2012; February 2 to February 11, 2012; and March 26 to March 30, 2012.

The LIRAa is Rapid Index Survey for *Aedes aegypti*—LIRAa—for Entomological Surveillance of *A. aegypti* in Brazil.

The LIRAa presents larval indices through the property infestation and Breteau indices and includes the types of containers (breeding sites). The percentage of positive houses given by the property infestation index (PII) is the result of the formula: PII = Positive Properties × 100/Properties Searched.^29^

The Breteau Index (BI) considers the positive recipients and properties surveyed without considering the productivity of breeding types. It is expressed by the following formula: BI = Positive Containers × 100/Properties Searched.^29^

The Container Type Index (ITR) is expressed by the formula: ITR = Positive Containers “X” × 100/Total Positive Containers. In this case “X” is the container type.^29^

In all the places, the *A. aegypti* infestation index rapid survey (LIRAa), which uses larval survey, and the use of ovitraps were carried out simultaneously for comparison of positive ovitrap index (POI) with the LIRAa.

In the surveys for the LIRAa, all containers with standing water were inspected in the intradomicile and peridomicile areas, where 1 in every 5 homes was investigated, using a larval net with 200 µm mesh with the help of a light source (flashlight). The captured larvae and pupae were transferred to flasks containing breeding water, and in the laboratory, the larvae, when necessary, were bred up to the third instar and then identified. Adults were obtained from the pupae for identification using the key contained in Walter Reed Biosystematics Unit (WRBU).^30^

Along with the LIRAa method, traps for oviposition were setup. The ovitraps^31^ consisted of black plastic pots with a capacity of 500 mL. A hardboard pallet, 15 × 3 cm (duratree) was placed inside the receptacle and 300 mL of hay infusion (30%) were added.

The hay infusion was prepared by the addition of 10 g of *Megathyrsus maximus* (Jacq.) B.K. Simon & S.W.L. Jacobs (Guinea grass) to 10 L of water, which was kept in a plastic drum covered with a screen and allowed to ferment for 7 days. The hay infusion to be used in the trap was filtered through a 200-µm mesh and diluted with tap water to obtain a 30% solution.

Of the homes sampled in the larval surveys, 60 were selected for installation of the ovitraps, where 1 was placed in the intradomicile and the other in the peridomicile area, totaling 120 traps in every survey. A week after installation, the traps were recovered, with their pallets duly labeled.

### Preference of oviposition (intradomicile or peridomicile)

**Figure 1.**
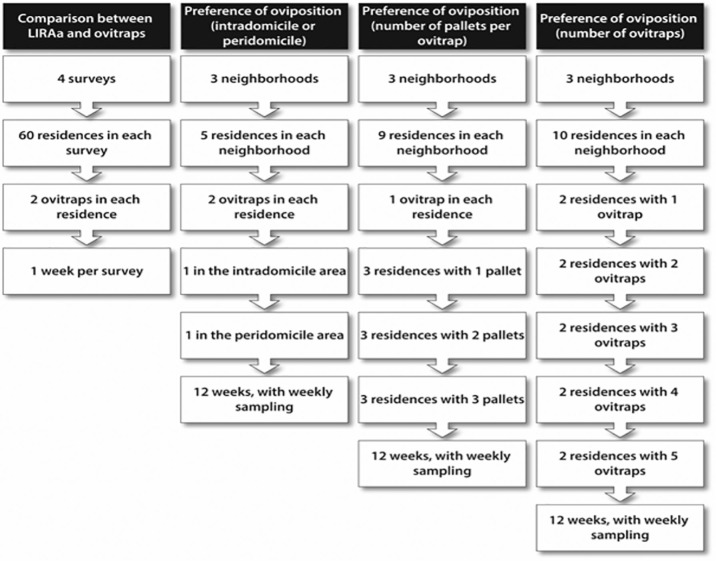
Sampling design for comparison between LIRAa and ovitraps and preference of oviposition by *Aedes aegypti* depending on the installation place, the number of ovitraps per residence, and the number of pallets per ovitrap.

Ovitraps were installed in the neighborhoods Cambé 4, Ana Rosa 3, and Ana Rosa 2 in the period of November 2011 to February 2012. In each locality, 5 homes were examined, 1 per block, where 2 ovitraps were installed, 1 inside and the other outside each home, totaling 15 sampling points and 30 traps installed (Figure 1). The ovitraps were left in place for 12 weeks, with weekly change of liquid in the trap and substitution of pallets.

### Preference of oviposition (number of pallets per ovitrap)

Nine homes were selected in each neighborhood, totaling 27 homes, distributed 1 per block, in the period of January 2012 to March 2012. The ovitraps were installed containing 1, 2, and 3 pallets, with 1 trap per home and varying the number of pallets (Figure 1). The maintenance of the traps and collection procedures were carried out as described above.

### Preference of oviposition (number of ovitraps)

Ten homes were selected in 3 localities (Cambé 4, Ana Rosa 3, and Ana Rosa 2), totaling 30, with 1 home on every block in these 3 localities. The survey was performed in the period of November 2011 to February 2012. Around 1 to 5 ovitraps were installed in these homes (Figure 1). The collection procedures and identification were done as described in section “Preference of oviposition (intradomicile or peridomicile).”

For all experiments using ovitraps, the eggs on each pallet were counted with a stereomicroscope (Opton, 10 to 40X) and later hatching took place in plastic trays, incubated at 25°C ± 2°C until reaching the fourth instar for specifies identification.

### Analysis of data

The rapid index surveys of *A. aegypti* by means of larval survey were analyzed using the BI and property infestation index (PII), as recommended by the Ministry of Health of Brazil.^32^ The first represents the number of infested containers with larvae for every 100 residences and the latter the proportion of houses infested with immature forms of *A. aegypti*. The oviposition in the ovitraps were analyzed via the POI, which indicates the percentage of traps that contained eggs, and by the egg density index (EDI), that shows how many eggs, on average, were found per trap.^33^ A simulated BI and PII were also calculated using the positive ovitraps as infested containers.

Comparative evaluation of the efficiency of larval survey and ovitraps in the surveillance of *A. Aegypti* density were carried out using the paired t-test in R v.3.6.0,^34^ between the BI and the simulated BI, as well as between the PII obtained from the 2 methods.

The indices used to analyze the oviposition in the ovitraps, POI and EDI, were used to evaluate the preference of oviposition by *A. aegypti* depending on the installation place, the number of ovitraps per residence, and the number of pallets per ovitrap, to evaluate the best configuration of ovitraps for surveillance of *A. aegypti* in areas of interest. The difference between the indices were evaluated in R v.3.6.0,^34^ using paired t-test for the installation place and 1-way repeated measures analysis of variance (ANOVA) or Friedman rank sum test for number of ovitraps and number of pallets.

## Results

A total of 5712 eggs were collected in the ovitraps installed during the 4 LIRAa surveys of *A. aegypti* conducted by agents of the Municipal Secretariat of Health (Table 1). The highest number of homes with traps positive for *A. aegypti* occurred in the third survey carried out in February 2012. Of the 60 homes inspected in the month of February, 52 (86.7%) showed mosquito eggs, and it was also when the highest number of eggs was recorded, 3526 (61.7%). Of these 4 collections, totaling 240 homes with ovitraps, 130 (54.2%) were positive with respect to presence of *A. aegypti* eggs, where 174 (36.3%) of the 480 traps checked during the study showed mosquito eggs.

**Table 1.** Larval survey and use of ovitraps installed in homes where LIRAa were carried out by the Secretary of Health of Cambé, Paraná, Brazil, from November 2011 to April 2012, in an urban area of Cambé, Paraná, Brazil.

	First collection (November)	Second collection (January)	Third collection (February)	Fourth collection (April)	Total
Ovitraps					
Positive homes	9	32	52	37	130
Positive traps	11	37	72	54	174
Number of eggs	151	425	3526	1610	5712
POI	9.2	30.8	60.0	45.0	36.3
EDI	13.7	11.5	49.0	29.8	32.8
BI based on ovitraps	18.3	61.7	120.0	90.0	72.5
PII based on ovitraps	15.0	53.3	86.7	61.7	54.2
LIRAa					
BI according to larval survey	1.7	0.0	3.3	3.3	2.1
PII according to larval survey	1	0	2	2	1.3

Abbreviations: POI, positive ovitrap index; EDI, egg density index; BI, Breteau index; PII, property infestation index.

**Figure 2.**
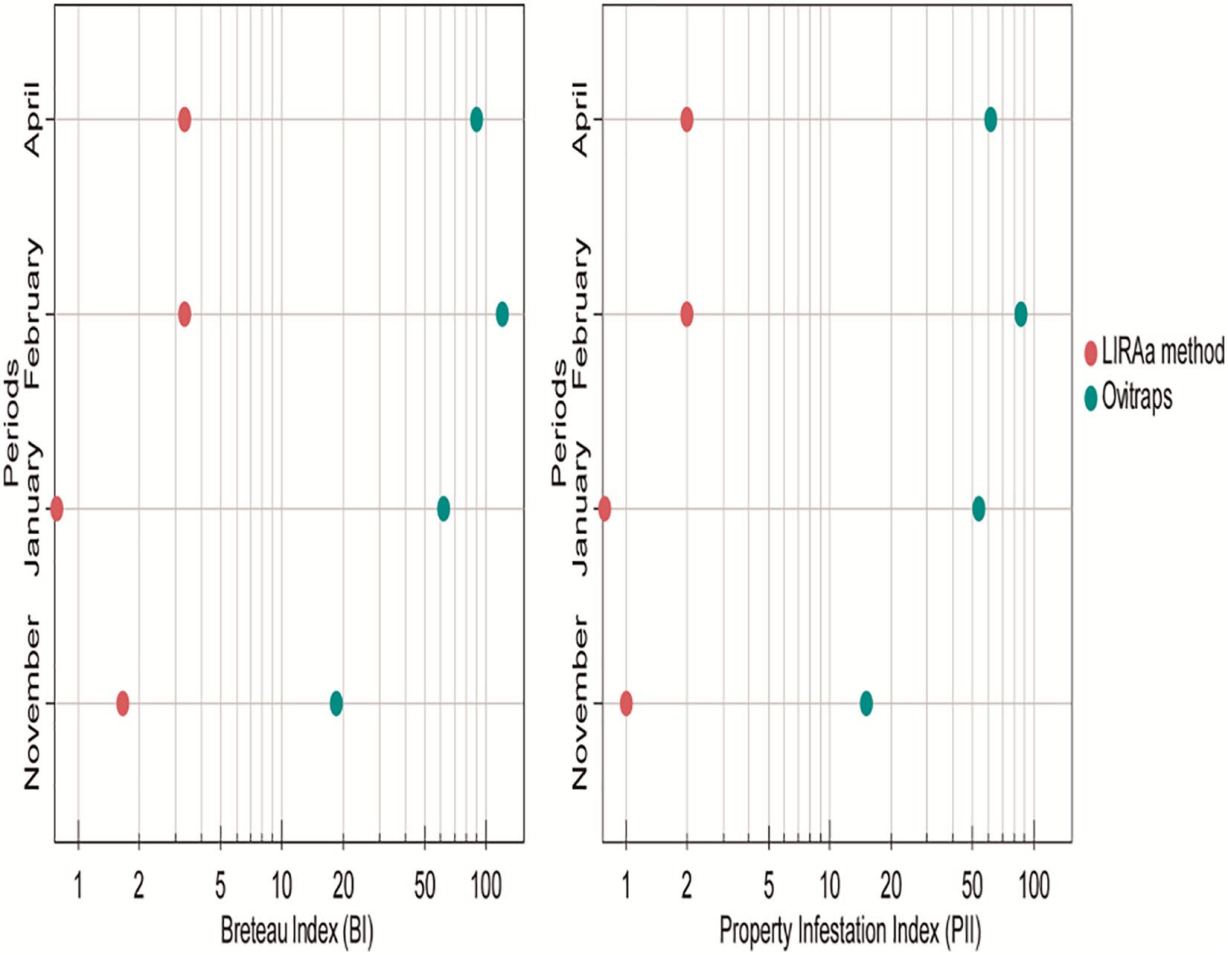
Comparison between the efficiency of ovitraps and larval survey in the Breteau index (BI) and the property infestation index (PII), from November 2011 to April 2012, in an urban area of Cambé, Paraná, Brazil.

Significant differences occurred during the 4 surveys (*P* = .045) between the BI using the larval survey and the ovitraps using the paired t-test (Figure 2). In the 4 repetitions, the BI was greater with the ovitraps when compared with the larval survey. The highest BI with the ovitraps was seen in the month of February (120), whereas this index using the larval survey was 3.33. In the January survey, the BI was 61.67 while no larva was found in the homes by the survey larval method (Table 1).

In all surveys, a higher number of homes with positive ovitraps was found when compared with the PII (Figure 2) according to the paired t-test (*P* = .036). The larval survey showed that 1.3% of the residences were positive for immature forms of *A. aegypti*, whereas 54.2% were positive by ovitraps (Table 1). Furthermore, in January, 53.3% of homes sampled with ovitraps showed the presence of *A. aegypti*, while the PII was zero.

In the experiment with traps installed in the intra- and peridomicile areas, followed for 12 weeks, POI and EDI were higher in the peridomicile, but without statistical difference (*P* = .074 and *P* = .866, respectively) using the paired t-test, although the number of eggs were more stable at the peridomicile (Table 2).

**Table 2.** Preference of oviposition of *Aedes aegypti* according to ovitrap location, number of pallets per ovitrap and number of ovitraps per residence, as measured by positive ovitrap index (POI) and egg density index (EDI).

	Ana Rosa 3		Ana Rosa 2		Cambé 4		General Index	
	POI	EDI	POI	EDI	POI	EDI	POI	EDI
Location								
Intradomicile	25.0	26.3	20.00	58.75	16.7	12.4	20.6	32.5
Peridomicile	45.0	40.3	26.7	31.88	30.0	33.8	33.9	35.4
Number of pallets								
1 pallet	11.1	25.7	51.9	14.57	51.9	23.3	38.3	21.2
2 pallets	29.6	100.5	14.8	2.25	14.8	8.8	19.8	37.2
3 pallets	22.2	31.8	37.0	8.30	37.0	8.3	32.1	16.1
Number of ovitraps								
1 ovitrap	12.5	18.3	8.3	2.00	16.7	7.5	12.5	9.3
2 ovitraps	20.8	33.5	8.3	8.00	14.6	21.3	14.6	20.9
3 ovitraps	13.9	17.8	11.1	10.38	8.3	13.8	11.1	14.0
4 ovitraps	12.5	27.6	4.2	20.25	8.3	45.0	8.3	30.9
5 ovitraps	10.0	40.2	8.3	30.10	10.0	9.6	9.4	26.6

Knowing that *A. aegypti* females jump around in carrying out oviposition, ovitraps were installed with different number of pallets. It was observed that the POI and EDI were highest in traps with 1 pallet in the neighborhoods Cambé 4 and Ana Rosa 2, but not in Ana Rosa 3, where traps with 2 pallets were higher. However, the differences were not significant according to the Friedman rank sum test (*P* = .717 and *P* = .717, respectively) (Table 2).

Also, it was necessary to determine the quantity of traps that would be ideal, in each home, for the evaluation of the population of *A. aegypti* in that area. The POI was highest in homes that contained 2 traps, but the differences did not show statistical significance (*P* = .291) according to the Friedman rank sum test and the EDI was highest in homes with 4 traps, although this difference was not statistically significant (*P* = .156) according to the 1-way repeated measures ANOVA (Table 2).

## Discussion

The sensitivity of ovitrap traps for the detection of the presence of *A. aegypti* was greater than that of the larval survey method. This finding reinforces the results previously obtained and reported in the literature. In many homes, the ovitrap traps showed positivity with respect to the presence of *A. aegypti*, while with the larval survey, it was not possible to detect the vector. But in 1988, Gomes affirmed that the infestation indices (property and Breteau) are weak indicators of the quantity of mosquitoes, and thus, limited for evaluating risks of disease transmission. Cardoso et al,^35^ in studies with ovitraps and BI in Catanduva-SP, after a dengue epidemic, observed that in the ovitraps the detection of *A. aegypti* occurred 2 months after control measures employed by the Ministry of Health, while by means of BI, they were detected after 4 months. The comparative studies of efficiency between the BI, PII, and ovitrap positivity index revealed the POI as the most efficient and sensitive detection method for *A. aegypti* in relation to larval indices.^13,20,23,36^

Ribeiro,^37^ besides proving the sensitivity of ovitraps for detecting the presence of the vector in places where larval survey was unable to detect it, demonstrated the importance of their use in indicating geographically the range of infestation in the country. According to Glasser and Gomes,^38^ the levels of larval infestation cannot, in particular circumstances, show a correlation with the incidence of dengue, where transmission has been recorded in periods with low BIs. Thus, the data obtained in this study corroborated these findings.

Besides the greater sensitivity for detecting the presence of mosquitoes in the observation area, the oviposition traps provided more information on the mosquito population. The use of ovitraps as sentinels in endemic areas increases the sensitivity of techniques for detecting the presence of the vector and can allow the implementation of preventive measures. Gama et al^39^ found greater sensitivity of ovitraps the larval survey, observing that PII and BI showed stable values during the whole experiment and that the POI varied 16.7% to 76.9%. Resende^40^ observed a greater sensitivity for entomological indices provided by ovitraps and MosquiTRAP than for larval survey, further showing that when the larval indices decline, the indices obtained in ovitraps and MosquiTRAP increase. Rueda^41^ found that the use of ovitrap indices is more advantageous than the Breteau and property indices, because it allows the mapping of the population density of *A. aegypti* in a locality. The ovitrap performs the early detection of vectors, besides the chemical control evaluation of adults, when the larval density indices do not detect, with rapidity and intensity, the density of the vector population.^42^ Therefore, the detection of eggs in the oviposition traps confirms the presence of gravid females in full hematophagous and consequently reproductive activity. Thus, in using ovitraps, it is important to characterize the early epidemiological situation to implement measures to prevent the expansion of dengue in a locality.

According to De Azevedo Marques et al,^43^ the larval infestation indices used are not sensitive for determining the levels of disease transmission. The immature forms’ detection in the larval survey cannot be a reflection of simultaneous presence of adult females in a locality, where these eggs can be accumulated in dry periods and hatch in response to favorable environmental conditions. The survey made by means of ovitraps estimates the population in current reproductive activity, allowing the monitoring and continuous evaluation of the population of *A. aegypti*.^44^

*Aedes aegypti* colonized the intradomicile and peridomicile areas, not demonstrating any preference for either of these environments in oviposition. Dhang et al,^45^ using ovitraps in residential areas in Kuala Lumpur, Malaysia, observed that there was no significant difference for *A. aegypti* between interior and exterior. According to Gomes et al,^46^ *A. aegypti* are found more often in intradomicile than peridomicile environments. Wan Norafikah et al,^47^ in studies with ovitraps in areas located in Malaysia, found that ovitrap positivity was greater in the peridomicile than intradomicile area. Wu et al,^48^ in studies with ovitraps in Southern Taiwan, found that more eggs of *A. aegypti* were collected inside than outside homes.

To determine the oviposition by *A. aegypti* females with respect to availability of substrate, it was observed that the use of only 1 pallet per ovitrap was sufficient for detecting the presence of *A. aegypti* and possibly estimating the population of females. In opposition to the results obtained, Acioli^44^ demonstrated that ovitraps with 3 pallets provided greater physical capacity for receiving higher numbers of eggs. This author also found that the maximum number of eggs in an ovitrap was 8925 eggs.

Also, differences were observed in the colonization of traps with 1 or more pallets, in the different locations studied, which can be related to the different levels of infestation of mosquitoes in these areas and not necessarily to the availability of greater number of pallets in the traps. Thus, the use of ovitraps with only 1 pallet is an adequate method for detecting the presence of *A. aegypti* in different areas, as previously demonstrated by Vezzani et al^49^ and Ríos-Velásquez et al.^50^

Traps with 1 pallet were more indicated because there was no significant difference between traps with different number of pallets. The increase in substrate for oviposition did not increase the efficiency of the oviposition trap for detecting the presence of the vector species.

On analyzing the POI obtained in homes with different number of traps, it was seen that 2 traps per home would be sufficient in monitoring projects in the urban area, even knowing the behavior of females of *A. aegypti* of using various places for oviposition in the same home.^51,52^

Besides the focus aimed at determining the presence of mosquitoes and offering a view of their population density in a particular area, the use of ovitraps can help in the control of the vector, by eliminating the eggs, more efficiently than only eliminating the breeding sites, as observed by Cheng et al.^53^

In a study carried out in areas with a high infestation of *A. aegypti*, where they found that the BI decreased significantly in areas with the presence of ovitraps. According to Marten,^54^ the use of ovitraps is an effective method for reducing the population of *A. aegypti*, where the ovitraps compete with breeding sites.

The results obtained in this study indicate that the indices calculated using the data obtained from the ovitraps showed greater sensitivity for detecting the presence and abundance of *A. aegypti*, and that 2 traps are sufficient at each surveillance point for use in conventional monitoring practices.

The use of only 1 pallet per trap can be indicated for surveillance works, because there was no significant difference between the traps with different number of pallets. The installation of traps only in the peridomicile area is sufficient for monitoring studies, thereby preventing difficulties in their installation and monitoring and making the process easier, requiring fewer hours for examining the ovitraps.

## References

[1] World Health Organization (WHO). Dengue and severe dengue. http://www.who.int/mediacentre/factsheets/fs117/es/. Updated 2019. Accessed January 19, 2019.

[2] Secretaria de Vigilância em Saúde (SVS). Plano de Contingência Nacional para a febre Chikungunya, Versão 2. Brasilia, Brazil: SVS; 2014.

[3] Zanluca C Melo VC Mosimann AL Santos GI Santos CN Luz K. First report of autochthonous transmission of Zika virus in Brazil. Mem Inst Oswaldo Cruz. 2015;110:569-572. 26061233 10.1590/0074-02760150192 PMC4501423

[4] Secretaria de Vigilância em Saúde (SVS). Boletim Epidemiológico. Monitoramento dos casos de dengue, febre de chikungunya e febre pelo vírus Zika até a Semana Epidemiológica 48. Vol. 46 Brasilia, Brazil: SVS; 2015.

[5] Chiaravalloti-Neto F de Moraes MS Fernandes MA. Avaliação dos resultados de atividades de incentivo à participação da comunidade no controle da dengue em um bairro periférico do Município de São José do Rio Preto, São Paulo, e da relação entre conhecimentos e práticas desta população. Cad Saúde Pública. 1998;14:S101-S109. 9700229

[6] Forattini OP. Culicidologia Médica. São Paulo, Brazil: Edusp; 2002.

[7] Díaz-González EE Kautz TF Dorantes-Delgado A, et al First report of *Aedes aegypti* transmission of chikungunya virus in the Americas. Am J Trop Med Hyg. 2015;93:1325-1329. 26416113 10.4269/ajtmh.15-0450 PMC4674253

[8] Secretaria de Vigilância em Saúde (SVS). Boletim Epidemiológico. Monitoramento dos casos de dengue, febre de chikungunya e febre pelo vírus Zika até a Semana Epidemiológica 16. Vol. 47 Brasilia, Brazil: SVS; 2016.

[9] Secretaria de Vigilância em Saúde (SVS). Informe Epidemiológico Nº 27—Monitoramento dos casos de Microcefalia no Brasil até a Semana Epidemiológica 48. Vol. 46 Brasilia, Brazil: SVS;2016.

[10] Marcondes CB. Entomologia Médica e Veterinária. São Paulo, Brazil: Ed Atheneu; 2001.

[11] Nogueira RMR Miagostovichi MP Schatzmayr HG, et al Dengue in the State of Rio de Janeiro, Brazil. Mem Inst Oswaldo Cruz. 1999;94:297-304. 10419380 10.1590/s0074-02761999000300004

[12] Barreto LM Teixeira MG Dengue no Brasil: Situação epidemiológica e contribuições para uma agenda de pesquisa. Estudos Avançados. 2008;22:53-72.

[13] Braga IA Gomes AC Nelson M Mello RCG Bergamaschi DP de Souza JMP Comparação entre pesquisa larvária e armadilha de oviposição, para detecção de Aedes aegypti. Rev Soc Brasil Med Trop. 2000;33:347-353. 10.1590/s0037-86822000000400003 10936947

[14] Vega-Rúa A Zouache K Girod R Failloux AB Lourenço-de-Oliveira R. High level of vector competence of *Aedes aegypti* and *Aedes albopictus* from ten American countries as a crucial factor in the spread of Chikungunya virus. J Virol. 2014;88:6294-6306. 24672026 10.1128/JVI.00370-14 PMC4093877

[15] Brasil. Plano Nacional de controle da dengue. Ministério Saúde Brasil. 2002;1: 1-34.

[16] Barcellos C Pustai AK Weber MA Brito MRV Identificação de locais com potencial de transmissão de dengue em Porto Alegre através de técnicas de geoprocessamento. Rev Soc Brasil Med Trop. 2005;38:246-250. 10.1590/s0037-86822005000300008 15895177

[17] Brasil. Casos de dengue: Brasil, Grandes regiões e Unidades Federadas—1990 a 2011. Brasilia, Brazil: Disponível No Ministério Da Saúde http://portal.saude.gov.br/portal/arquivos/pdf/casos_de_dengue_classica_brasil_1990_201.pdf. Updated 2012. Accessed December 20, 2012.

[18] Ai-Leen TG Song RJ. The use of GIS in ovitrap monitoring for dengue control in Singapore. Dengue Bull. 2000;24:110-116.

[19] Polson KA Curtis C Seng CM Olson JG Chantha N Rawlins SC. The use of ovitraps baited with hay infusion as a surveillance tool for *Aedes aegypti* mosquitoes in Cambodia. Dengue Bull. 2002;26:178-184.

[20] Morato VCG da Glória Teixeira M Gomes AC Bergamaschi DP Barreto ML Infestação por *Aedes aegypti* estimada por armadilha de oviposição em Salvador, Bahia. Rev Saúde Púb. 2005;39:553-558. 10.1590/s0034-89102005000400006 16113903

[21] Santos RC. Atualização da distribuição de *Aedes albopictus* no Brasil. Rev Saúde Púb. 2003;37:671-673. 14569346

[22] Balestra RAM Pereira RKO Ribeiro MJS Silva JS Alencar J Ocorrência de *Aedes (Stegomyia) albopictus* Skuse em Área Urbana do Estado do Tocantins. Neotrop Entomol. 2008;37:233-235. 18506306 10.1590/s1519-566x2008000200020

[23] Regis L Monteiro AM Melo-Santos MA, et al Developing new approaches for detecting and preventing *Aedes aegypti* population outbreaks: basis for surveillance, alert and control system. Mem Inst Oswaldo Cruz. 2008;103:50-59. 18368236 10.1590/s0074-02762008000100008

[24] Nunes LS Trindade RBT Souto RNP Avaliação da atratividade de ovitrampas a *Aedes (Stegomyia) aegypti* Linneus (Diptera: Culicidae) no bairro Hospitalidade, Santana, Amapá. Biota Amazônia Open J Syst. 2011;1:26-31.

[25] Rawlins SC Martinez R Wiltshire S Legall G. Comparison of surveillance systems for the dengue vector *Aedes aegypti* in Port of Spain, Trinidad. J Am Mosq Contr Assoc. 1998;14:131-136. 9673912

[26] Masuh H Seccacini E Zerba E Licastro SA. *Aedes aegypti* (Diptera: Culicidae): monitoring of populations to improve control strategies in Argentina. Parasitol Res. 2008;103:167-170. 18344071 10.1007/s00436-008-0945-0

[27] Chadee DD Corbet PS Talbot H. Proportions of eggs laid by *Aedes aegypti* on different substrates within an ovitrap in Trinidad, West Indies. Med Veterin Entomol. 1995;9:66-70. 10.1111/j.1365-2915.1995.tb00118.x 7696690

[28] Ipardes. Perfil do município de Cambé, Paraná. http://www.ipardes.gov.br/perfil_municipal/MontaPerfil.php?Municipio=86180. Updated 2012. Accessed October 4, 2012.

[29] Ministério da Saúde. Levantamento Rápido de Índices para Aedes aegypti—LIRAa para vigilância—entomológica do Aedes aegypti no Brasil: metodologia para avaliação dos índices de Breteau e Predial e tipo de recipiente. Brasilia, Brazil http://bvsms.saude.gov.br/bvs/publicacoes/levantamiento_rapido_indices_aedes_aegypti_liraa.pdf. Updated 2015. Accessed May 3, 2018.

[30] Walter Reed Biosystematics Unit (WRBU). Mosquito identification resources. http://www.wrbu.org/VecID_MQ.html. Updated 2019. Accessed January 20, 2019.

[31] Fay RW Eliason DA. A preferred oviposition site as a surveillance method for Aedes aegypti. Mosq News. 1966;26:531-535.

[32] Brasil. Diagnóstico rápido dos municípios para a vigilância entomológica do *Aedes aegypti* no Brasil—LIRAa. Ministério Saúde Brasil. 2005;1:1-61.

[33] Gomes AC. Medidas dos níveis de infestação urbana para *Aedes aegypti* e *Aedes albopictus* na infestação do Estado de São Paulo. Iesus. 1998;1:49-56.

[34] R Core Team. R: A Language and Environment for Statistical Computing. Vienna: R Foundation for Statistical Computing http://www.R-project.org/. Updated 2019 Accessed January 5, 2018.

[35] Cardoso RP Jr Scandar SAS de Mello NV Ernandes S Botti MV Nascimento EMM Detecção de *Aedes aegypti* e *Aedes albopictus* na zona urbana do município de Catanduva-SP, após controle de epidemia de dengue. Rev Soc Brasil Med Trop. 1997;30:37-40. 9026829

[36] Silva JCS Avaliação da aplicação de novos métodos de monitoramento populacional na vigilância entomológica em dengue no Município do Ipojuca, Pernambuco. Monografia (Especialização em Gestão de Sistemas e Serviços de Saúde), Centro de Pesquisa Aggeu Magalhães, Fundação Oswaldo Cruz, Rio de Janeiro, Brazil; 2010.

[37] Ribeiro CMN Facilidades e limitações observadas durante a implantação de novas metodologias para o controle da dengue no município de Santa Cruz do Capibaribe—Pernambuco. Dissertação (Mestrado Profissional em Saúde Pública), Centro de Pesquisas Ageu Magalhães, Fundação Oswaldo Cruz, Recife, Brazil; 2010.

[38] Glasser CM Gomes AC. Clima e Sobreposição da Distribuição de *Aedes aegypti* e *Aedes albopictus* na Infestação do Estado de São Paulo. Rev Saúde Pública. 2002;36:166-172. 12045797 10.1590/s0034-89102002000200008

[39] Gama RA Silva EM Silva IM Eiras AE. Avaliação da MosquiTRAP na detecção de *Aedes (Stegomyia) aegypti* (L.) (Diptera: Culicidae) durante estação seca em Belo Horizonte, MG. Neotrop Entomol. 2007;36:294-302. 17607465 10.1590/s1519-566x2007000200018

[40] Resende MC. Estudo multicêntrico do uso da armadilha MosquiTRAP para captura de Aedes aegypti e geração de índices de vigilância entomológica. Tese (Doutorado em Ciências Biológicas—área de concentração em Entomologia), Universidade Federal de Minas Gerais, Belo Horizonte, Brazil; 2009.

[41] Rueda BZ. Comparação da eficácia dos métodos “Índice de Breteau” e armadilha de oviposição (ovitrampas) na obtenção dos índices de infestação de Aedes (Stegomyia) aegypti e Aedes (Stegomyia) albopictus no Município de Botucatu, SP. Dissertação (Mestrado em Biologia Geral), Universidade Estadual Paulista, Instituto de Biociências, Botucatu, Brazil; 2009.

[42] De Azevedo Marques CC Marques GRDAM de Brito M dos Santos Neto LG de Campos Ishibashi V de Assis Gomes F. Estudo comparativo de eficácia de larvitrampas e ovitrampas para vigilância de vetores de dengue e febre amarela. Rev Saúde Pública. 1993;27:237-241. 8209154 10.1590/s0034-89101993000400002

[43] Ordóñez-Gonzalez JG Mercado-Hernandez R Flores-Suarez AE Fernández-Salas I. The use of sticky to estimate dispersal of *Aedes aegypti* in northeastern Mexico. J Am Mosq Contr Assoc. 2001;17:93-97. 11480827

[44] Acioli RV. O uso de armadilhas de oviposição (ovitrampas) como ferramenta para monitoramento populacional do Aedes spp em bairos do Recife. Dissertação (mestrado em saúde publica), Centro de Pesquisas Aggeu Magalhães, Fundação Oswaldo Cruz, Recife, Brazil; 2006.

[45] Dhang CC Benjamin S Saranum MM, et al Dengue vector surveillance in urban residential and settlement areas in Selangor, Malaysia. Trop Biomed. 2005;22:39-43. 16880752

[46] Gomes A de Souza JMP Bergamaschi D, et al Atividade antropofílica de *Aedes aegypti* e *Aedes albopictus* em área sob controle e vigilância. Rev Saúde Pública. 2005;39:206-210. 15895139 10.1590/s0034-89102005000200010

[47] Wan Norafikah O Nazni WA Noramiza S, et al Ovitrap surveillance and mixed infestation of *Aedes aegypti* (Linnaeus) and *Aedes albopictus* (Skuse) in Northern Region and Southern Region of Malaysia. Health Environ J. 2011;2:15.

[48] Wu HH Wang CY Teng HJ, et al A dengue vector surveillance by human population-stratified ovitrap survey for *Aedes* (diptera: Culicidae) adult and egg collections in high dengue-risk areas of Taiwan. J Med Entomol. 2013;50:261-269. 23540112 10.1603/me11263

[49] Vezzani D Velázquez SM Schweigmann N. Seasonal pattern of abundance of *Aedes aegypti* (Diptera: Culicidae) in Buenos Aires City, Argentina. Mem Inst Oswaldo Cruz. 2004;99:351-356. 15322622 10.1590/s0074-02762004000400002

[50] Ríos-Velásquez CM Codeço CT Honório NA, et al Distribution of dengue vectors in neighborhoods with different urbanization types of Manaus, state of Amazonas, Brazil. Mem Inst Oswaldo Cruz. 2007;102:617-623. 17710307 10.1590/s0074-02762007005000076

[51] Da Silva VC Scherer PO Falcão SS, et al Diversidade de criadouros e tipos de imóveis frequentados por *Aedes albopictus* e Aedes aegypti. Rev Saúde Pública. 2006;40:1106-1111. 17173170 10.1590/s0034-89102006000700021

[52] Da Silva Vieira GS Lima SC. Distribuição Geográfica da dengue e índice de infestação de *Aedes aegypti* em Uberlândia (MG), 2000 a 2002. Caminhos Geog. 2006;11:107-122.

[53] Cheng M Beng-Chuan H Barnetnett RE Goodwin N. Role of a modified ovitrap in the control of *Aedes aegypti* in Houston, Texas, USA. Bull World Health Organ. 1982;60:291-296. 6980740 PMC2535964

[54] Marten GG. Using ovitraps to assess the quantity of mosquito larval habitat during local eradication with source reduction and ovitraps. J Med Entomol. 2012;49:640-646. 22679872 10.1603/me10284

[55] Secretaria de Vigilância em Saúde (SVS). Boletim Epidemiológico. Monitoramento dos casos de arboviroses urbanas transmitidas pelo Aedes (dengue, chikungunya e Zika), Semanas Epidemiológicas 1 a 34. Vol. 50 Brasilia, Brazil: SVS; 2019 http://portalarquivos2.saude.gov.br/images/pdf/2019/setembro/11/BE-arbovirose-22.pdf

